# Advancing Early Warning Systems for Malaria: Progress, challenges, and future directions - A scoping review

**DOI:** 10.1371/journal.pgph.0003751

**Published:** 2025-05-14

**Authors:** Donnie Mategula, Judy Gichuki, Karen I. Barnes, Emanuele Giorgi, Dianne Janette Terlouw

**Affiliations:** 1 Department of Clinical Sciences, Liverpool School of Tropical Medicine, Liverpool, United Kingdom; 2 Malawi Liverpool Wellcome Programme, Blantyre, Malawi; 3 Kamuzu University of Health Sciences, Blantyre, Malawi; 4 Institute of Healthcare Management, Strathmore University, Nairobi, Kenya; 5 Division of Clinical Pharmacology, Department of Medicine, University of Cape Town, Cape Town, South Africa; 6 Centre for Health Informatics, Computing and Statistics, Lancaster University, Lancaster, United Kingdom; PLOS: Public Library of Science, UNITED STATES OF AMERICA

## Abstract

Malaria Early Warning Systems (EWS) are predictive tools that often use climatic and other environmental variables to forecast malaria risk and trigger timely interventions. Despite their potential benefits, the development and implementation of malaria EWS face significant challenges and limitations. We reviewed the current evidence on malaria EWS, including their settings, methods, performance, actions, and evaluation. We conducted a comprehensive literature search using keywords related to EWS and malaria in various databases and registers. We included primary research and programmatic reports on developing and implementing Malaria EWS. We extracted and synthesized data on the characteristics, outcomes, and experiences of Malaria EWS. We screened 6,233 records and identified 30 studies from 16 countries that met the inclusion criteria. The studies varied in their transmission settings, from pre-elimination to high burden, and their purposes, ranging from outbreak detection to resource allocation. The studies employed various statistical and machine-learning models to forecast malaria cases, often incorporating environmental covariates such as rainfall and temperature. The most common mode used is the time series model. The performance of the models was assessed using measures such as the Akaike Information Criterion (AIC), Root Mean Square Error (RMSE), and adjusted R-squared (R ^2^). The studies reported actions and responses triggered by EWS predictions, such as vector control, case management, and health education. The lack of standardized criteria and methodologies limited the evaluation of EWS impact. Our review highlights the strengths and limitations of malaria early warning systems, emphasizing the need for methodological refinement, standardization of evaluation metrics, and real-time integration into public health workflows. While significant progress has been made, challenges remain in automating forecasting tools, ensuring scalability, and aligning predictions with actionable public health responses. Future efforts should enhance model precision, usability, and adaptability to improve malaria prevention and control strategies in endemic regions.

## Introduction

The fight against malaria has reached a critical phase. Despite remarkable strides in reducing the global malaria burden from 2000 to 2015, progress has stalled since 2016, especially in high-burden countries within sub-Saharan Africa [1–3]. This stagnation signals an urgent need for innovative tools and strategies to revive the push toward the World Health Organization’s (WHO) 2030 targets for malaria elimination [4]. For these elimination targets to not merely be aspirational, there is a need to deploy new or effective vector control measures, diagnostic tools, antimalarial medications, and social behaviour change communication. A transformation of malaria surveillance systems is equally essential, shifting from passive reporting to dynamic systems capable of tracking hotspots, forecasting outbreaks, and evaluating the effectiveness of interventions [5].

Malaria Early Warning Systems (EWS) are embedded within these enhanced surveillance systems, which are critical for forecasting and mitigating potential outbreaks. By synthesizing data on intervention strategies, environmental conditions, and resistance patterns, EWS equips health authorities and policymakers with the means to respond effectively to upcoming threats. This proactive approach is vital for reducing the strain on healthcare infrastructure and saving lives. As countries move closer to malaria elimination, the frequency of outbreaks is expected to increase due to the ‘heterogeneous’ nature of transmission [5,6]. This heterogeneity, driven by disparities in intervention uptake, climate variability, and resistance among vectors and parasites, necessitates robust EWS to navigate and control the evolving landscape of malaria transmission [7].

An effective malaria EWS is a powerful predictive tool, enabling public health officials, governments, and stakeholders to take informed, pre-emptive actions to prevent impending outbreaks. Integrating data on intervention uptake, climatic conditions, vector and parasite resistance, and other critical factors is essential [8]. However, the implementation and development of EWS face significant challenges. Diverse methodologies to predict future malaria risk present two distinct scenarios: some EWS provide reasonable certainty (reliability associated with the warning information) but inadequate lead time (duration between issuing a warning or alert and the onset of the event) for action. In contrast, others offer good lead time but with modest certainty in predictions. Striking a balance between accurate forecasts and timely response is complex yet crucial in developing effective malaria EWS [9,10].

The sustainability of investments in malaria EWS is a significant challenge, with funding often being reactive to an outbreak or disaster. This pattern can undermine the long-term effectiveness and maintenance of these systems. For an EWS to be successfully integrated into malaria surveillance, there is a need for improved infrastructure, capacity building, and collaboration among stakeholders, including the community, researchers, and policymakers. These improvements are crucial for the effective functioning and utilization of EWS in malaria prevention and control efforts [11].

The Roll Back Malaria (RBM) initiative established a framework for malaria EWS in 2001, guiding its development and implementation [12]. However, progress in establishing EWS in Africa has been slow over the past two decades. The role and importance of malaria EWS vary depending on the epidemiological setting. In low-burden settings aiming for elimination, EWS can help identify transmission hotspots and optimize resource allocation for targeted interventions [13].

In contrast, in high-burden settings, EWS are critical for anticipating seasonal surges and ensuring adequate case management and vector control preparedness. Research has shown that malaria incidence is closely linked to seasonal variations, with higher burdens observed during warmer and wetter seasons, underscoring the necessity for EWS to predict these trends. Despite these differences, the fundamental objective remains to provide timely and actionable predictions that enhance malaria control and prevention strategies. However, many existing models have not been explicitly designed to accommodate these epidemiological variations, limiting their practical utility across diverse settings. A scoping review identified that while various models exist, their applicability varies across transmission settings, from pre-elimination to high burden, indicating a need for adaptable EWS frameworks.

Zinszer et al.‘s (2012) systematic review of malaria EWS focused on forecasting methodologies, predictors, and model evaluations but did not address other critical aspects, such as actions following early warning predictions, performance evaluation, and integration into existing systems for scalability and sustainability [14]. More recent reviews have similarly been limited in scope. Hussain-Alkhateeb et al. (2021) examined EWS across multiple vector-borne diseases. Still, they did not focus on malaria-specific forecasting approaches or their integration into health systems[8]. Baharom et al. (2022) focused on climate-driven malaria projections [15]. Still, they did not assess the broader methodological landscape or the implementation challenges of EWS in different epidemiological settings. These gaps emphasize the need for a thorough review, synthesizing malaria-specific early warning system methodologies while assessing their practical application, effectiveness, and usefulness.

Given the continued burden of malaria and the increasing risks posed by climate variability and changing epidemiological patterns, a comprehensive assessment of malaria EWS that evaluates their methodological approaches, implementation challenges, and practical applications is still needed. Recent studies have emphasized the importance of data-driven malaria prediction models, highlighting the role of climate, environmental, and socio-economic factors in outbreak prediction [14,16,17]. However, the translation of these models into operational EWS remains inconsistent.

This review aims to synthesize current evidence on malaria EWS, examining their settings, EWS methodologies, performance assessment approaches, and response actions. Unlike previous reviews, our study systematically assesses the strengths and weaknesses of existing EWS, explores their application across different malaria-endemic settings, and evaluates their impact on decision-making in malaria control.

In this review, we set out to answer the following questions.

1. What are the transmission settings in which malaria EWS are developed?
2. What are the methodologies used in developing malaria EWS?
3. What actions have been documented following malaria EWS predictions?
4. What are the approaches to evaluating a malaria EWS’s performance, effect, and impact?

## Methods

This review focused on developing and implementing malaria EWS for populations affected by or at risk of malaria, including all age groups and demographics. These systems, either standalone or integrated into broader programs, utilized routinely collected data or data from studies and surveillance systems to predict future malaria risks. Instead of comparing these systems with specific alternatives, we assessed various characteristics of each malaria EWS. The outcomes evaluated included predictions or forecasts of malaria cases, disease, mortality, and anti-malarial drug resistance. We incorporated original research published in peer-reviewed journals and available online programmatic reports.

### Eligibility criteria

The studies included met the following inclusion and exclusion criteria.

#### Inclusion criteria.

- Original research in a peer-reviewed journal - Published or available through online programmatic reports.
- Develops a prediction model for predicting malaria cases, deaths, or anti-malarial drug resistance.
- Malaria is the disease of interest. Specific outcomes include malaria cases, disease, death, and antimalarial drug resistance.
- Presents the development, evaluation, or other experiences of an EWS in a standalone setting or as part of a program.

#### Exclusion criteria.

- Studies focusing solely on general malaria trends, risk factors, or predictors without examining or forecasting the specific outcomes related to EWS.
- Studies focusing on malaria in non-human subjects, such as animal or *in vitro* studies.
- Any study that does not explicitly address a prediction model, including the development, evaluation, or other experiences with EWS.
- Studies that do not present predictions or forecasted outcomes for malaria cases, disease, death/mortality, or antimalarial drug resistance.
- Non-peer-reviewed apart from online programmatic reports.

#### Information sources.

We used the EBSCOhost platform, which gave us access to several significant databases: Medline Complete, Global Health, CNHL Complete, and Green File.

#### Search strategy.

The final search presented in the manuscript was done on 28 January 2024.

### Keywords

“malaria” as Medical Subject Headings (MeSH) terms and as a keyword

AND

“early warning” OR “prediction” OR “forecasting” as MeSH terms

As accessible text terms truncated as follows predict* OR forecast* OR “early warn*”

#### Limiters.

- Published between January 2012 and January 2024 (a prior review was done before 2012 [14]).
- Online full text available.
- Peer-reviewed.
- Human.

There were no language restrictions in the search

### Data management

#### Study selection.

The Rayyan tool was used to manage the extracted studies. Two reviewers (DM and JG) independently screened all retrieved studies’ titles and abstracts and excluded irrelevant studies based on eligibility criteria. Full-text screening was conducted for all potentially eligible studies to assess eligibility based on the inclusion and exclusion criteria. Any disagreements between reviewers were resolved through discussion.

#### Data extraction and management.

Data extraction used a pre-designed standard data extraction form developed in-house. This form included vital information such as the author(s), year of publication, study location, study year, study setting, study design, and EWS description parameters.

### Risk of bias assessment

The risk of bias in the included studies was not formally assessed because the scope of this review did not encompass a complete systematic approach, which typically necessitates such an evaluation. However, inclusion and exclusion criteria were strictly applied to maintain the integrity and reliability of the findings.

### Data synthesis

The synthesis of data from the included studies was structured to provide a comprehensive understanding of malaria EWS. This process included a summary of the characteristics of the studies, including the methodology, population, geographic location, and primary findings. A thorough narrative synthesis was done to align with the specific objectives of the review, providing an in-depth analysis and synthesis of key findings. These included: (1) Exploration of the settings and methods used in developing each malaria EWS, data sources, prediction modeling, and technological platforms, (2) Operational aspects of the malaria EWS, focusing on effectiveness, user experience, implementation challenges, and sustainability in diverse contexts, (3) Actions and responses triggered by EWS predictions with identification of successful practices, challenges, and coordination among various stakeholders, and (4) Review of methodologies and criteria used to evaluate EWS’ performance, effect, and impact.

## Results

**Fig 1:**
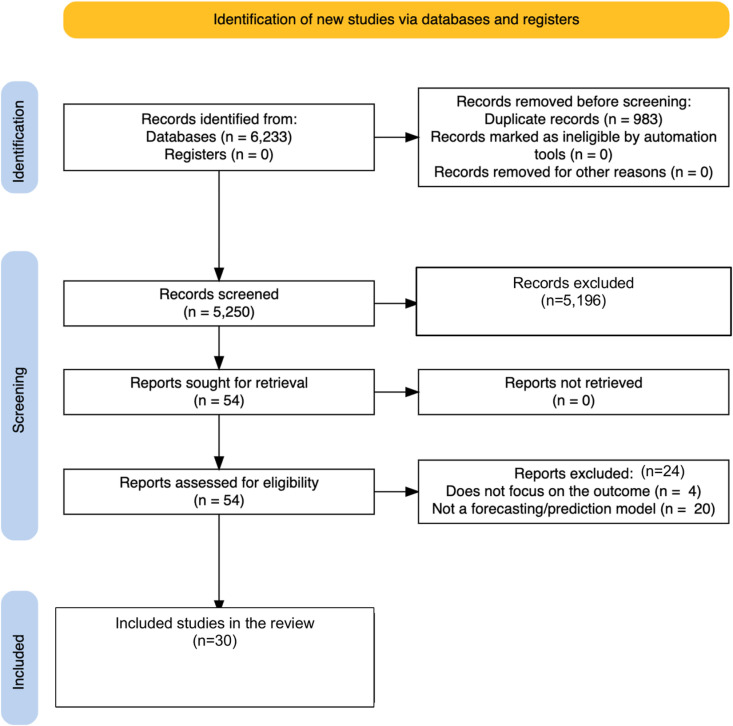
Review Flow diagram.

Following our search, we retrieved 6,233 database records (Table 1). No additional records were identified from the programmatic reports’ registers. After 983 duplicate records were removed, titles and abstracts of the remaining 5250 records were screened. After excluding ineligible articles, we identified 54 studies for a detailed full-text evaluation of their eligibility. During the full-text review, we excluded 24 studies that did not meet the inclusion criteria, with 30 included. The reasons for exclusion have been included in the PRISMA diagram (Fig 1). All identified studies were in English apart from one study in Mandarin that did not meet the titles and abstract review criteria.

**Table 1:** Number of studies identified through each database search (including duplicates).

Database	Number of studies identified
Medline Complete	4399
Global health	1291
CNHL Complete	473
Green File	70
Others	0

**Fig 2:**
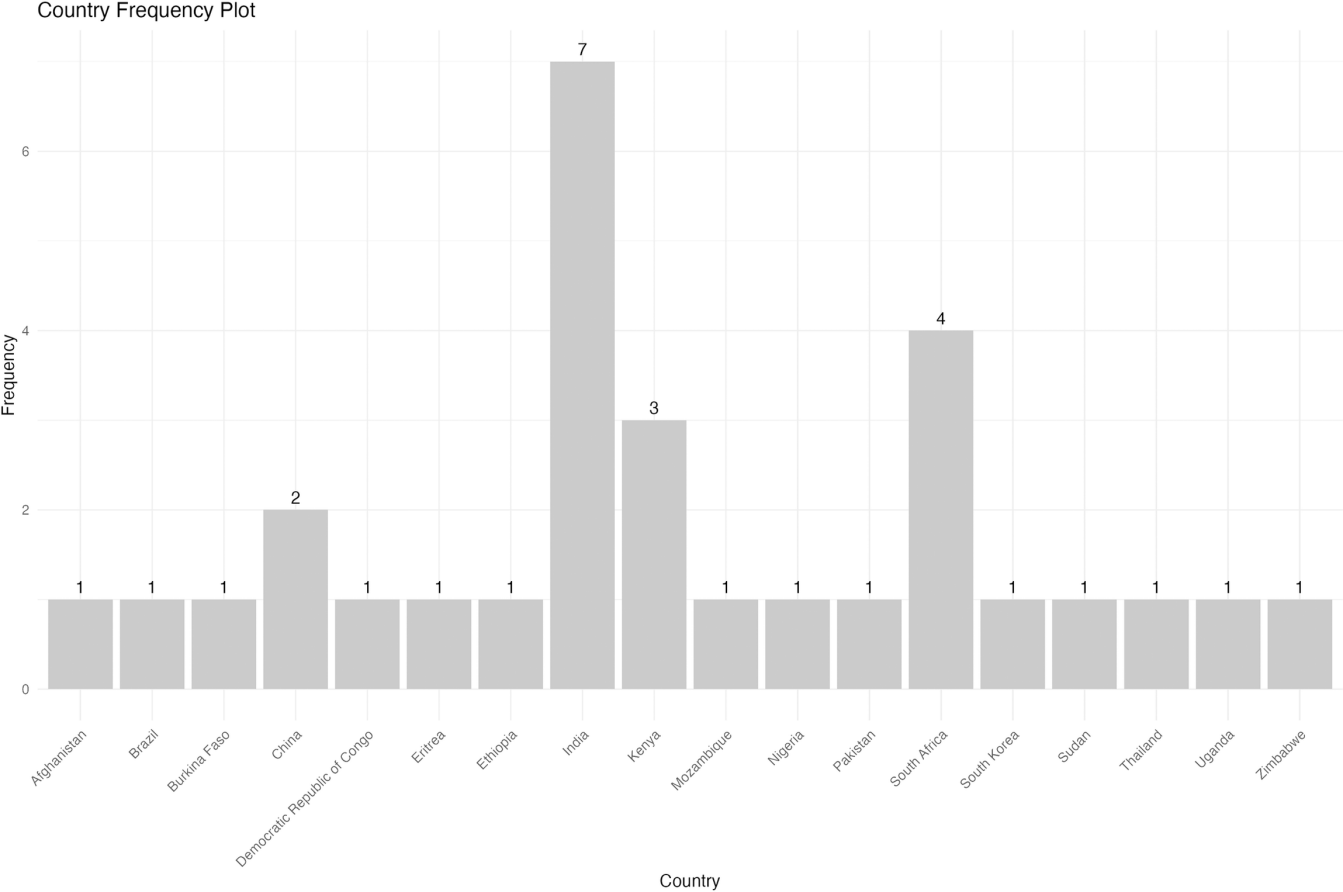
Frequency plot showing the countries where included studies were conducted.

Supplementary File 1 (S1 Table) contains all collated data. The review identified studies from 18 countries, with India contributing the most significant proportion, accounting for seven studies (23%). Sixteen of the thirty included studies (53%) were conducted in Africa, with South Africa and Kenya being the most represented, as illustrated in Fig 2.

### Settings where malaria EWS were developed

**Fig 3:**
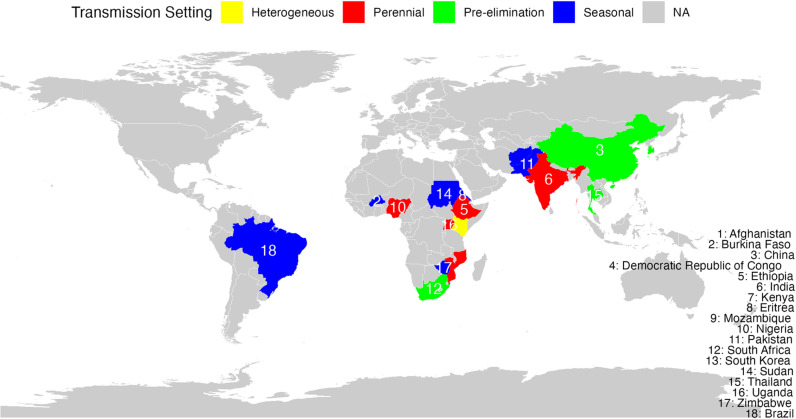
Countries where the included studies were done and their malaria transmission setting.

Our systematic review identified a range of papers on malaria EWS across varied transmission settings. These settings included seasonal transmission areas such as Afghanistan (1/30, 3%) [18] and Pakistan [19], where EWS models predicted malaria trends based on climatic fluctuations. Other included studies were from highly heterogeneous regions like Ethiopia [20] and Kenya [21], where the EWS focused on the high transmission settings of the countries. Several studies (7/30, 23.3%) from India [22–28] and Mozambique [29,30] represented perennial transmission settings, demonstrating year-round prediction systems. Four of the included studies (13.3%) covered regions nearing malaria elimination, such as South Africa [31–34], or focused on detecting residual transmission in Thailand [35]. Other aspects included regions with high transmission during rainy seasons [36], provinces with malaria epidemics in China [37], areas with stable transmission in Uganda[38], and those with low risk in South Korea [39]. Some studies focused on specific [34]challenges, such as the re-emergence of malaria in border regions of China [40] or leveraging indigenous knowledge in high-risk zones in Zimbabwe [41] (Fig 3).

Fig 3 was generated using computer code and derivative works from the geoBoundaries

project (https://www.geoboundaries.org) under a CC BY 4.0 license with permission from Runfola, D. et al. (2020) [42].

### Approaches to developing EWS

Several approaches were used to develop malaria EWS (Table 2). We broadly classified the models into statistical, machine learning, and indigenous knowledge models. Statistical approaches typically relied on predefined models where covariates were carefully selected based on prior knowledge and were explicitly included in the model structure to explain the relationship between variables. These models often assumed a specific distribution for the data and emphasized interpretability, allowing for clear inferences about the effects of each covariate. In contrast, machine learning approaches usually focused on predictive performance rather than interpretability, using algorithms that could automatically select, transform, and weigh covariates in complex ways without requiring prior assumptions about the data’s distribution. While statistical models typically conveyed uncertainty through confidence intervals and p-values, machine learning models often relied on techniques such as cross-validation and bootstrapping to estimate uncertainty. However, such estimates were not always directly interpretable.

**Table 2:** Approaches to the development of EWS.

Approach Category	Methods Used	Studies/References
Statistical	Correlation	Dhiman et al (2017)
Statistical	Dynamic System	Harris et al (2020)
Statistical	Geostatistical model	Colborn et al (2018)
Statistical	Process	Roy et al (2015)
Statistical	Regression	Bouma et al (2016), Sewe (2017), Verma (2018)
Statistical	Time series	Adeola et al (2014), Anwar et al (2016), Ebhuoma et al (2016), Hussien et al (2017), Karuri et al (2018), Kifle et al (2019), Kumar et al (2020), Mopuri et al (2023), Riaz et al (2023), Wang et al (2023), Zinszer et al (2023)
Statistical	Time series, Multimodel	Panzi et al (2022)
Statistical	Time series, nonlinear	YoonHee et al (2019)
Machine Learning	General Machine Learning	Mohapatra et al (2021)
Machine Learning	Neural Networks	Barboza et al (2016), Haddawy et al (2020), Kamana et al (2022), Santosh et al (2022)
Machine Learning	Rule based	Buczak et al (2015), Martineau et al (2022)
Machine Learning	Supervised Learning	Harvey et al (2021), Brown et al (2020)
Indigenous Knowledge	Community EWS	Macherera et al (2016)

Twenty of the thirty included studies (67%) used statistical models to develop EWS, with 13 out of the 20 (65%) studies using a time series analysis [18,19,21,24,28,31,32,34,37,38,43–45]. The time series analysis approaches included AutoRegressive Integrated Moving Average (ARIMA) in three studies (15%) [21,28,43]. In contrast, some studies used variations of ARIMA, such as the Seasonal AutoRegressive Integrated Moving Average (SARIMA) in four of the 20 studies (20%) [24,31,34,44] and Seasonal AutoRegressive Integrated Moving Average with Exogenous Regressors (SARIMAX) to account for seasonal adjustments and exogenous variables in one study [18]. Other studies integrated various statistical methods, as Panzi et al. (2022) demonstrated, who employed a multi-model approach to construct the malaria EWS model [45].

Other statistical approaches included using regression models in three of the twenty studies (15%) to quantify the relationship between variables [20,27,46]. In contrast, one study used a correlation model to identify the strength of associations [22]. One study applied dynamic systems theories, such as the theory of critical slowing down, to anticipate shifts in malaria transmission under varying conditions [47]. In comparison, process-based models were employed in one study to simulate the complex interactions between environmental factors and malaria dynamics, providing insights into how malaria may evolve [25].

Nine of the identified studies (30%) utilized machine learning models for EWS development, with two of the nine studies (22%) applying supervised learning methods, including Gaussian Processes and Random Forests, or a combination of generalized linear models (GLM), ensemble methods (EM), and support vector machines for data-driven predictions in dense populations [29,36]. Neural networks were utilized in four of the nine studies (44%), with Bayesian networks used in one of the four studies [35]. In contrast, Long Short-Term Memory (LSTM) models were applied in three studies for their effectiveness in handling large datasets and complex patterns, such as climate change effects and city-specific malaria trends [26,40,48]. In two studies, additional machine learning approaches included using rule-based methods, including the fuzzy association rule mining classifier and machine learning classification models [33,39]. In contrast, one study used a general machine approach (Waikato environment for knowledge analysis - WEKA) for classifier selection [23].

One study, the Gwanda District study in Zimbabwe, utilized Indigenous Knowledge Systems for their malaria EWS [41].

### Conveying uncertainty of predictions

Seven studies incorporated measures of uncertainty in their predictions, primarily using confidence intervals [18,21,25,30,34,38,45]. Anwar et al. (2016) predicted malaria cases in Afghanistan from January 2014 to September 2015, providing confidence intervals to express the uncertainty in their forecasts. Similarly, Roy et al. (2015), Karuri et al. (2016), Ebhuoma et al. (2018), and Zinszer et al. (2015) included confidence intervals in their predictions [21,25,34,38]. Panzi et al. (2022) used confidence intervals in forecasting malaria cases in the Democratic Republic of the Congo (DRC) from 2020 to 2030, while Colborn et al. (2018) employed non-exceedance probabilities, an alternative method for representing predictive uncertainty [30,45]. This distinction highlights the different approaches to quantifying and communicating uncertainty across these studies. The models that did not include uncertainty in their predictions mostly used machine-learning models.

### Covariates and data sources

A range of covariates were used in the included studies (Table 3). Standard covariates across the studies included environmental factors such as rainfall, temperature, and humidity, alongside vegetation indices like normalized difference vegetation index (NDVI) that measures vegetation health and density and enhanced vegetation index (EVI), which is helpful in areas with dense vegetation. Out of the 30 included studies, 16 (53%) had rainfall or precipitation covariates, 14 (47%) used temperature, and 5 (17%) included humidity variables (Table 3). One study considered climate indices like the Oceanic Niño Index (ONI) [22]. Two studies included demographic data [26,29]. Seven out of the 30 studies (23%) did not include covariates, relying solely on malaria case reports (Table 3). Additionally, the identified studies utilized various data sources, including public health reports, national disease control databases, health facility data, and satellite-derived data (Table 3).

**Table 3:** The covariates included in the models and data sources.

Year	Author	Country	Covariates	Data Source (malaria incidence data)
2014	Kumar et al [29]	India	Rainfall, temperature, wind Speed, humidity	Public Health Data (2006–2013)
2015	Roy et al [25]	India	Rainfall, population	National Institute of Malaria Research Data
2015	Zinszer et al [38]	South Korea	Rainfall, temperature, vegetation, clinical data	Health Facility & Satellite-Derived Data
2015	Buczak et al[39]	Uganda	Rainfall, Temperature, social data, intervention data	Korea Centers for Disease Control and Prevention website for the period from 2004–2013.
2016	Anwar et al [18]	Afghanistan	Rainfall, NDVI, EVI, NDWI	Ministry of Public Health Reports (2005–2015)
2016	Bouma et al [20]	Ethiopia	Sea surface temperature over the Pacific and Indian Oceans	Malaria Case Reports (1982–2005)
2016	Karuri et al [21]	Kenya	Rainfall	Pediatric Malaria Admission Data (1990–2011)
2016	Haddaway et al[35]	Thailand	Environmental variables, time lagged effects	Community Reports & Environmental Data
2016	Macherera et al[41]	Zimbabwe	Insects, plant phenology, animals, weather, cosmological indicators	Community Reports & Indigenous Environmental Indicators
2017	Dhiman et al [18]	India	Monthly Oceanic Nino Index (ONI)	CBHI & NVBDCP Data (1994–2015)
2017	Sewe et al [46]	Kenya	LSTs (Day and night), precipitation	Siaya District Hospital Admission Data (2003–2013)
2017	Hussien et al [43]	Sudan	None used	Routine Incidence Data
2018	Verma et al [27]	India	Not included	Data obtained via Google Search
2018	Colborn et al [30]	Mozambique	Not included	NMCP Routine Data
2018	Ebhuoma et al [34]	South Africa	Rainfall, Temperature, Wind Speed, Humidity	Health Facility & Satellite-Derived Data
2019	Wang et al [37]	China	Temperature, humidity, air pressure,vapor pressure, moisture level, wind velocity, precipitation, sunshine duration, days with daily precipitation	Yunnan prProvince mMalaria dData (2011–2017)
2019	Kifle et al [44]	Eritrea	Rainfall	Malaria Incidence Data (2012–2016)
2019	Adeola et al [31]	South Africa	Rainfall, NDVI, EVI, NDWI	Malaria Case Observations (2013–2017)
2019	Kim YoonHee et al and Ratnam [32]	South Africa	Temperature, precipitation	Malaria Case Data in Vhembe, Limpopo (1998–2015)
2020	Mopuri et al [24]	Nigeria	Rainfall, temperature, NDVI	NVBDCP, Visakhapatnam Data (2001–2016)
2020	Santosh et al[26]	India	Temperature, rainfall, age, sex, vegetation index	RoutineEpidemiological Data (1995–2018)
2020	Harris et al [47]	Kenya	Not included	Hospital Case Reports (1965–2002)
2020	Brown et al[29]	India	Demographics, temperature, rainfall	Hospital Routine Data
2021	Harvey et al [36]	Burkina Faso	Not included	Integrated e-Diagnostic Approach (IeDA) Database
2021	Mohapatra et al [23]	India	Rainfall, temperature, humidity, topography	Directorate of Public Health Services, Odisha Data (2002–2017)
2022	Kamana et al [40]	Democratic Republic of Congo	Temperature	Chinese Centre for Disease Control and Prevention
2022	Panzi et al[45]	China	Rainfall, temperature, humidity, wind speed	DRC Epidemiological Surveillance Directorate Database
2022	Martineau et al [33]	South Africa	Sea surface temperature over the Pacific and Indian Oceans	Malaria Institute, Tzaneen Data (1998–2020)
2022	Barboza et al [48]	Brazil	Not included	Not included
2023	Riaz et al[19]	Pakistan	Not included	MOH Routine Data

NDVI: Normalized Difference Vegetation Index

EVI: Enhanced Vegetation Index

NDWI: Normalized Difference Water Index

IeDA: Integrated e-Diagnostic Approach

DRC: Democratic Republic of the Congo

LST: Land Surface Temperature

CBHI: Community-Based Health Insurance

NVBDCP: National Vector Borne Disease Control Programme

NMCP: National Malaria Control Program

MOH: Ministry of Health.

### EWS model performance methodology

Table 4 below summarises the EWS assessment methods used. Commonly used metrics for assessing performance were the Root Mean Square Error (RMSE) [18,19,23,24,30,37,40,46], Mean Absolute Error (MAE) [24,29,40,46] and R Squared (R^2^) values [20,25,28], alongside more complex statistical tools like the Akaike Information Criterion (AIC)[18,19,24] and Bayesian Information Criterion (BIC) [44]. Some studies focused on precision measures such as accuracy, sensitivity, and specificity [25,26,33,36,39]. In contrast, three studies utilized correlation or auto functions and error estimation methods [18,27,45].

**Table 4:** Assessing the performance of the EWS models.

Year	Author	Methods for Assessing Performance
2014	Kumar et al [28]	R Squared (R^2^), ACF
2015	Roy et al [25]	Accuracy, R Squared (R^2^)
2015	Buczak et al [39]	Model Positive Predictive Value (PPV) and Sensitivity: 0.842 and 0.681
2015	Zinszer et al [38]	Not specified
2016	Anwar et al [18]	Autocorrelation function, Akaike Information Criterion (AIC), Root Mean Square Error (RMSE), Adjusted R2
2016	Bouma et al [18]	R Squared (R^2^)
2016	Karuri et al[21]	AIC, Root Mean Squared Error of Estimation (RESE), ACF
2016	Haddawy et al [35]	Not specified
2016	Macherera et al [41]	Not specified
2017	Dhiman et al [18]	Not specified
2017	Sewe et al [46]	RMSE, MAE
2017	Hussien et al [43]	AIC, MAE
2018	Verma et al [27]	Correlation
2018	Colborn et al [30]	RMSE
2018	Ebhuoma et al [34]	Standardized Mean Square Error (SMSE), Spearman’s correlation
2019	Wang et al [37]	RMSE, Mean Absolute Scaled Error (MASE), Mean Absolute Deviation (MAD)
2019	Kifle et al [44]	R Squared (R^2^), Bayesian Information Criterion (BIC)
2019	Adeola et al [31]	Adjusted R^2^
2019	Kim YoonHee et al and Ratnam [32]	Specificity, Sensitivity, RMSE
2020	Mopuri et al [24]	RMSE, Mean Absolute Percentage Error (MAPE), MAE, R^2^, AIC
2020	Santosh et al [26]	Accuracy, Sensitivity, Precision
2020	Harris et al [47]	Not specified
2020	Brown et al [29]	MAE, Mean Squared Error (MSE)
2021	Harvey et al [36]	Two-tailed precision
2021	Mohapatra et al [23]	Root Mean Square Error (RMSE), Accuracy, Kappa, Receiver Operating Characteristics (ROC) Value
2022	Kamana et al [40]	RMSE, Mean Absolute Error (MAE)
2022	Panzi et al [45]	MASE, Autocorrelation Function (ACF), The Box-Pierce test
2022	Martineau et al [33]	Accuracy, Specificity, Sensitivity, Precision
2022	Barboza et al [48]	Not specified
2023	Riaz et al [19]	RMSE, MAPE, MAE, R^2^, AIC

Overall, the models utilized in the included studies demonstrated strong predictive capabilities. This assessment was made by examining the authors’ comments on their metrics. Details of the final evaluations can be found in supplement document(S1 Table)For example, Yoon-Hee et al. (2019) distributed a lag non-linear time series malaria prediction model for cases in South Africa that showed good performance, particularly for short-term predictions of 1–2 weeks ahead, achieving correlation coefficients greater than 0.8 Although the accuracy of the predictions decreased with increased lead time, the model still performed well up to 16 weeks in advance [32]. In Kifle et al. ‘s (2019) SARIMA-based model in Eritrea, monthly malaria case predictions from 2012 to 2016 closely aligned with observed cases. Minor discrepancies were, however, noted in the third quarter of the fourth year and the first quarter of the fifth year [44]. Martineau et al. (2022) machine learning forecasting models in South Africa achieved an accuracy rate of 80% for predictions extending up to three seasons (nine months) ahead [33].

### Outbreak detection

Five studies (16.7%) utilized EWS for outbreak detection, employing varied methodologies [25,30,36,41,47]. Harvey et al. (2021) defined a malaria outbreak occurrence as the point at which the case rate surpassed the five-year mean for the same period plus two standard deviations, providing a statistically significant signal of an outbreak [36]. Roy et al. (2015) utilized a binary classifier to predict large outbreaks, defining an outbreak occurrence as the point when the probability exceeded a set threshold, which was then validated against actual data [25]. Harris et al. (2020) determined outbreaks by a substantial increase in cases, specifically when the count exceeded the previous months’ numbers by more than two and a half times. Lastly, Colborn et al. (2018) used exceedance probabilities (EPs) of relative risk to define outbreak thresholds, offering a probabilistic approach to outbreak detection [30].

### Actions following early warning predictions and incorporation into routine practice

Two studies (6.7%) extended malaria EWS development beyond initial creation and included a report on incorporating the EWS into routine practice. In Burkina Faso, Harvey et al. (2021) successfully integrated their malaria EWS into the district-level routine practice, streamlining the outbreak detection and response process, including the distribution of bed nets, indoor residual spraying, and larviciding [36]. Similarly, the EWS approach by Macherera et al. (2016) at the ward level in Zimbabwe demonstrated the system’s adaptability and effectiveness in local settings, improving community awareness and facilitating education campaigns. The district health teams incorporated the ward health teams into the malaria control plans [41].

## Discussion

The importance of malaria forecasting within the public health area cannot be overstated. The origins of malaria EWS can be traced to rudimentary forecasting methods pioneered by health practitioners in the 1900s who forecasted malaria using weather data [49]. These have evolved into more sophisticated models to support control and elimination efforts[8]. Our review identified 6,233 records published after January 2012. We included 30 studies that have enriched our understanding of malaria early-warning systems. Through these findings, we lay the groundwork for assessing the current practices and gaps within malaria forecasting and EWS development.

Forecasting methods should be scrutinized for their underlying assumptions, strengths, and weaknesses, with accuracy evaluations conducted on out-of-sample data. We acknowledge numerous forecasting methods, but it is of value to leverage standard forecasting measures to enable cross-study comparisons. Our review found that time series forecasting methods, especially regression-based approaches, are the most commonly used due to their flexibility and intuitive nature. However, these methods have limitations, such as overlooking serial autocorrelation in errors. This oversight can lead to biased estimates of predictor effects and underestimated standard errors. Therefore, it is essential to examine the residuals of such models for autocorrelation [50] We also identified studies that use ARIMA models, which can manage serial autocorrelation in the data, with their extended variants like SARIMA and ARIMAX providing additional predictive and forecast capabilities. However, these models require a substantial amount of data and examination of residuals to avoid misleading cross-correlation functions, which still need to be manually done in some cases [51]. Other complex methods in the machine-learning space have also been used. Studies reviewing the use of machine learning models in malaria EWS show their versatility across various ecosystems and capability to achieve greater accuracy. However, they point to the need for standardization to allow for assessment across models [52].

The current landscape of malaria forecasting is quite strong, mainly due to a solid foundation in methodology. Looking ahead, the focus should shift towards enhancing the performance of these models, refining their user interface, and automating their functions to facilitate their adoption by stakeholders within malaria-affected countries. To this end, it is essential to prioritize the development of intuitive platforms that can be seamlessly integrated into existing health systems’ workflows. An example is the EPIDEMIA system used in the Amhara region of Ethiopia, which utilizes near-real-time environmental data and patient records to provide updated malaria risk maps and forecasts. Such systems allow health officials to make timely decisions and improve intervention strategies based on current data rather than historical trends [50,53].

There is also a clear need to streamline these models to operate with (near) real-time data, enabling dynamic responses to evolving malaria trends. This could include developing adaptive algorithms that learn and improve from each prediction cycle, thereby increasing the accuracy and reliability of the forecasts. We also think fostering open-source communities around these models can accelerate innovation, allowing for collective problem-solving and sharing of best practices [54].

Another critical area is customizing these models to account for local environmental variables, socio-economic factors, and intervention strategies, which are crucial determinants of malaria transmission. As we move forward, it is also essential to consider the scalability of these models, ensuring they can be deployed in various settings, from rural clinics to national public health centers.

We must also invest in capacity building, providing training and support to local health practitioners and decision-makers so that they can effectively leverage these tools. This approach will increase the reach of malaria EWS and empower local actors to take charge of malaria mitigation efforts in their communities [55].

In addition to refining existing malaria forecasting models, there is an urgent need to expand the EWS scope to predict the incidence of malaria and critical outcomes like mortality rates and the emerging threats of antimalarial drug or insecticide resistance [56]. We found this to be a crucial gap in our review. The capacity to forecast these outcomes would be a significant leap forward, enabling health systems to allocate resources for immediate case management and long-term strategic planning. Predictive models that can, for example, anticipate the spread of drug-resistant strains could inform more effective malaria treatment policy decisions and guide research into new treatments. This broadening of focus will ensure that forecasting models remain relevant and potent tools in the evolving landscape of malaria control and prevention efforts.

The review found few documented actions following early warning predictions for malaria. These actions could range from mobilizing public health resources and the preemptive distribution of anti-malarial medications and bed nets to targeted vector control measures such as indoor residual spraying and larviciding. Community awareness and education campaigns could also be often intensified to improve prevention and early treatment-seeking behaviors [36,41].

Despite the recognized importance of malaria EWS, the literature needs to be more extensive in terms of their evaluations of their performance, effect, and impact after implementation. Such evaluations are necessary to ascertain the true efficacy of these systems. With assessment protocols and outcome data, refining EWS, tailoring them to specific environments, and justifying their adoption within routine health practices becomes easier. It is also essential to establish a standardized operational definition of an outbreak [57]. The diverse definitions used across studies make it challenging to compare methods or determine which is more effective [58]. This variability arises from the different approaches to defining an outbreak, whether it be a specific threshold of cases, expert judgment, or complex data models. Without a standard definition, each study may define an outbreak differently, leading to inconsistencies in model evaluation and interpretation [60].

While this review provides the landscape of malaria EWS, several limitations must be acknowledged. First, our search was limited to studies published after January 2012, following a prior systematic review by Zinszer et al [14]. While this ensures an updated synthesis of EWS methodologies, it excludes earlier work that might provide additional context on the evolution of these systems. Future research could integrate pre-2012 studies to offer a more comprehensive historical perspective. Second, the review was limited to peer-reviewed studies and programmatic reports accessible in selected databases. There may be relevant unpublished or non-indexed reports that were not captured. Additionally, we did not perform a formal risk-of-bias assessment, which limits our ability to evaluate the methodological rigor of individual studies. Finally, while our synthesis identifies key challenges in malaria EWS, the heterogeneity in study designs, performance metrics, and data sources makes direct comparisons difficult. Standardized reporting and evaluation frameworks are needed to enhance the comparability of findings across studies.

This review builds on previous reviews of malaria forecasting models, each with distinct focuses and limitations. Zinszer et al. (2012) [14] primarily examined forecasting methodologies and predictor variables but did not comprehensively assess actions following early warnings or integration into routine surveillance systems. More recent reviews, such as Hussain-Alkhateeb et al. (2021) [8], explored EWS across multiple vector-borne diseases, including malaria, but did not focus specifically on malaria forecasting models. Baharom et al. (2022) examined climate-driven malaria projections but did not assess implementation challenges and operational utility[15]. Compared to these reviews, our study systematically evaluates malaria-specific EWS regarding methodologies, performance metrics, and response actions. Our findings highlight the need for improved standardization in EWS assessment, better integration into health systems, and more substantial evidence of their real-world impact.

A significant ongoing debate in the literature concerns the reliability of climate data and its role in malaria forecasting. While climate variables such as temperature, rainfall, and humidity are widely used in malaria EWS, data quality, resolution, and availability inconsistencies can affect prediction accuracy. Satellite-derived climate data, for example, often require ground validation, and discrepancies in measurement techniques can lead to forecasting errors [44,53].

Additionally, while associations between climate covariates and malaria incidence are well-documented, establishing causality remains challenging. Many studies assume a direct link between climatic factors and malaria transmission, yet other factors such as intervention coverage, population movement, and socioeconomic conditions can modify these relationships[28,33]. Future work should integrate multi-factorial models that account for both climate and non-climate drivers of malaria transmission [23,45].

Malaria EWS must balance prediction accuracy with lead time. Exact models with short lead times may not allow timely responses, while models with longer lead times often carry more significant uncertainty[53,59]. Time series models, such as ARIMA and SARIMA, are widely used for short-term forecasting but struggle with long-term predictions in highly variable transmission settings[18,44]. Machine learning models offer improved accuracy but often lack interpretability and require substantial computational resources, limiting their practical application in low-resource settings [23,33]. Hybrid and ensemble approaches may help optimize both lead time and certainty, enhancing the usability of EWS in malaria-endemic regions [36,45]

## Conclusion

This review summarizes current evidence on malaria early warning systems, focusing on methodologies, performance metrics, and response actions. While time series and machine learning models are frequently used, challenges exist in balancing prediction accuracy with lead time and integrating these models into routine health systems. The lack of standardized frameworks to assess EWS effectiveness and concerns about climate data reliability further complicate the situation. For malaria forecasts to be actionable, they must be accurate, have proper spatial and temporal resolution, and consider the operational context, including data availability and technical skills. Different forecasting methods on the same datasets and expanding predictor variables will help refine these models. The future of malaria forecasting relies on improving model precision, usability, and automation while ensuring accessibility for health professionals in malaria-affected regions.

## Supporting information

S1 Table Table of included manuscripts data. (CSV)
